# The effectiveness of non-pyrethroid insecticide-treated durable wall lining to control malaria in rural Tanzania: study protocol for a two-armed cluster randomized trial

**DOI:** 10.1186/s12889-016-3287-3

**Published:** 2016-07-25

**Authors:** George Mtove, Joseph P. Mugasa, Louisa A. Messenger, Robert C. Malima, Peter Mangesho, Franklin Magogo, Mateusz Plucinski, Ramadhan Hashimu, Johnson Matowo, Donald Shepard, Bernard Batengana, Jackie Cook, Basiliana Emidi, Yara Halasa, Robert Kaaya, Aggrey Kihombo, Kimberly A. Lindblade, Geofrey Makenga, Robert Mpangala, Abraham Mwambuli, Ruth Mzava, Abubakary Mziray, George Olang, Richard M. Oxborough, Mohammed Seif, Edward Sambu, Aaron Samuels, Wema Sudi, John Thomas, Sophie Weston, Martin Alilio, Nancy Binkin, John Gimnig, Immo Kleinschmidt, Peter McElroy, Lawrence H. Moulton, Laura Norris, Trenton Ruebush, Meera Venkatesan, Mark Rowland, Franklin W. Mosha, William N. Kisinza

**Affiliations:** 1 National Institute for Medical Research, Amani Research Centre, Muheza, Tanzania; 2 Department of Disease Control, London School of Hygiene and Tropical Medicine, London, UK; 3 US President’s Malaria Initiative, Atlanta, GA USA; 4 Malaria Branch, Division of Parasitic Diseases and Malaria, Center for Global Health, Centers for Disease Control and Prevention, Atlanta, Georgia USA; 5 Kilimanjaro Christian Medical College, Moshi, Tanzania; 6 National Institute for Medical Research, Headquarters, Dar es Salaam, Tanzania; 7 Brandeis University, Heller School, Waltham, Massachusetts USA; 8 PMI Africa Indoor Residual Spraying Project, Abt Associates, London, UK; 9 Phoenix Ordinary LLC, Bridgewater, New Jersey USA; 10 President’s Malaria Initiative, United States Agency for International Development, Washington DC, USA; 11 Translating Research into Action Project (TRAction) University Research Co., LLC, Bethesda, Maryland USA; 12 Department of International Health, Johns Hopkins Bloomberg School of Public Health, Baltimore, Maryland USA

**Keywords:** Insecticide-treated wall lining, Long-lasting insecticidal nets, Malaria control, Cluster randomized controlled trial, Entomological inoculation rate, Insecticide resistance management

## Abstract

**Background:**

Despite considerable reductions in malaria achieved by scaling-up long-lasting insecticidal nets (LLINs) and indoor residual spraying (IRS), maintaining sustained community protection remains operationally challenging. Increasing insecticide resistance also threatens to jeopardize the future of both strategies. Non-pyrethroid insecticide­treated wall lining (ITWL) may represent an alternate or complementary control method and a potential tool to manage insecticide resistance. To date no study has demonstrated whether ITWL can reduce malaria transmission nor provide additional protection beyond the current best practice of universal coverage (UC) of LLINs and prompt case management.

**Methods/design:**

A two-arm cluster randomized controlled trial will be conducted in rural Tanzania to assess whether non-pyrethroid ITWL and UC of LLINs provide added protection against malaria infection in children, compared to UC of LLINs alone. Stratified randomization based on malaria prevalence will be used to select 22 village clusters per arm. All 44 clusters will receive LLINs and half will also have ITWL installed on interior house walls. Study children, aged 6 months to 11 years old, will be enrolled from each cluster and followed monthly to estimate cumulative incidence of malaria parasitaemia (primary endpoint), time to first malaria episode and prevalence of anaemia before and after intervention. Entomological inoculation rate will be estimated using indoor CDC light traps and outdoor tent traps followed by detection of *Anopheles gambiae* species, sporozoite infection, insecticide resistance and blood meal source. ITWL bioefficacy and durability will be monitored using WHO cone bioassays and household surveys, respectively. Social and cultural factors influencing community and household ITWL acceptability will be explored through focus-group discussions and in-depth interviews. Cost-effectiveness, compared between study arms, will be estimated per malaria case averted.

**Discussion:**

This protocol describes the large-scale evaluation of a novel vector control product, designed to overcome some of the known limitations of existing methods. If ITWL is proven to be effective and durable under field conditions, it may warrant consideration for programmatic implementation, particularly in areas with long transmission seasons and where pyrethroid-resistant vectors predominate. Trial findings will provide crucial information for policy makers in Tanzania and other malaria-endemic countries to guide resource allocations for future control efforts.

**Trial registration:**

NCT02533336 registered on 13 July 2014.

## Background

Recent, massive scale-up of long­lasting insecticidal net (LLINs) delivery and indoor residual spraying (IRS) has resulted in considerable progress in malaria control across sub-Saharan Africa [1, 2]. While both interventions can significantly reduce malaria burden when used alone [3, 4] or in combination [5], maintaining year-round or longer-term community protection remains operationally challenging. Hot dry seasons often deter net usage [6–8] and routine household damage may compromise the protective efficacy of LLINs despite their insecticidal longevity [9, 10]. The residual activities of current insecticides approved for IRS range from 3 to 12 months [11], rendering it logistically demanding and economically unsustainable in many endemic regions [12]. Furthermore, increasing mosquito insecticide resistance threatens to jeopardize the long­term effectiveness of both strategies [13–15].

Insecticide­treated wall lining (ITWL) may represent an alternate or complementary method of vector control and a potential tool to manage insecticide resistance. The currently available product consists of a high­density polypropylene fabric impregnated with a proprietary mixture of two classes of non­pyrethroid insecticides, which are designed to migrate differentially to sustain bioefficacy for a minimum of 3 years. This material can be fixed to the inner walls of houses and function as a long­lasting insecticidal reservoir. It is anticipated that ITWL will act in a similar manner to IRS, by reducing the longevity of indoor resting mosquitoes and overall vector population density (‘mass population effect’), if applied at high community-level coverage. Theoretically, ITWL could mitigate some of the limitations of existing control strategies. Once installed, household protection would be passive and not contingent on nightly behavioral compliance, unlike LLINs, and the longer lifespan of ITWL may circumvent the costs associated with repeated rounds of IRS, particularly in settings with year round transmission [16].

Initial studies of ITWL using insecticide-treated plastic sheeting (ITPS) demonstrated high mosquito mortality and reductions in malaria incidence in temporary labour shelters and refugee camps [17–20]. Subsequent, experimental hut trials of both pyrethroid and non-pyrethroid ITPS compared with LLINs have presented more equivocal results, which have been attributed to varying levels of insecticide resistance among vector populations [21–27]. The only community-level trial to evaluate carbamate-treated ITPS in combination with LLINs in an area of pyrethroid resistance in Benin reported no additional protection against malaria, potentially due to limited wall coverage and residual activity of the carbamate applied [28].

To date, existing data to support bioefficacy, feasibility and household acceptability of commercial pyrethroid ITWL are limited to small-scale trials conducted by the manufacturer and associates [29–32]. No controlled study has yet demonstrated that non-pyrethroid ITWL can reduce malaria burden nor provide additional protection beyond the current best practice of universal coverage (UC) of LLINs and prompt artemisinin combination therapy (ACT). Here we describe the study design and methodology of a two-armed cluster randomized controlled trial to evaluate the effectiveness of ITWL and UC of LLINs compared to UC of LLINs alone, on incidence of malaria infection among children in a rural area of Tanzania.

## Study objectives

### Primary objective

To assess the effectiveness of ITWL + LLINs compared to LLINs alone, on cumulative incidence of malaria parasitaemia (asymptomatic and symptomatic) in children aged 6 months to 11 years old.

### Secondary objectives

#### Epidemiological

- To assess the effectiveness of ITWL + LLINs compared to LLINs alone, on time to first episode of malaria (asymptomatic and symptomatic) in children aged 6 months to 11 years old.
- To assess the effectiveness of ITWL + LLINs compared to LLINs alone, on mean haemoglobin concentration in children aged 6 to 59 months old.

#### Entomological

- To assess the effectiveness of ITWL + LLINs compared to LLINs alone, on the entomological inoculation rate (EIR) of host seeking malaria vectors collected indoors and outside.
- To assess the effectiveness of ITWL + LLINs compared to LLINs alone, on the relative abundance of outdoor host-seeking *Anopheles gambiae* sensu lato and to measure any changes in indoor and outdoor biting.
- To assess the effectiveness of ITWL + LLINs compared to LLINs alone, on host feeding preferences of *An. gambiae* s. l.
- To compare changes in levels of insecticide resistance among *An. gambiae* s. l. populations in response to the use of two non-pyrethroid insecticides in ITWL + LLINs compared to LLINs alone.
- To monitor ITWL durability and insecticide bioavailability over a 12 month period.

#### Household acceptability

- To identify social, cultural and other factors that may affect acceptability of ITWL compared to LLINs in the study area.
- To assess perceptions regarding the implementation of ITWL among household members, community leaders, health workers and ITWL installers.

#### Socio-economic

- To estimate the incremental effectiveness of ITWL across the entire age spectrum.
- To determine the cost-effectiveness of ITWL in combination with other malaria control interventions (LLINs or IRS).

## Methods/design

### Study area and participant recruitment

The study site is situated in Muheza District, Tanga Region of northeastern Tanzania (5° S, 39° E), encompassing an area of approximately 4922 km^2^, ranging from a coastal plain at sea level to the Usambara Mountains at 1500 m. The climate is tropical with dense rainforest cover over the mountain region. Muheza District has four administrative divisions comprising 33 wards with 135 villages, mainly inhabited by subsistence farmers. The population of Muheza District was 204,461 residents in 2012 with an annual growth rate of 2.2 %; average household size was 4.3 individuals [33]. Across 60 villages, selected as candidate study clusters, a baseline survey conducted in January 2014, enumerated 92,692 individuals living in 23,977 households, with most homes constructed from mud or cement with palm thatch or metal roofs.

In Muheza, malaria transmission occurs throughout the year with two seasonal peaks following the long rainy season from July to August and the short rainy season from December to January. The main malaria vector species are *An. gambiae* sensu stricto*, An. arabiensis* and *An. funestus* [34, 35]; *Culex quinquefasciatus* is also abundant in the area [36, 37]. Significant resistance of local *An. gambiae* s. l. to pyrethroid insecticides (deltamethrin, lambdacyhalothrin and permethrin) has recently been reported and is expected to spread across this region [38, 39]. Historically, Muheza District has not been subjected to IRS but did receive LLINs from the nationwide UC campaign in 2011–12 and the under 5 campaign which preceded it [40]. During baseline surveys in 2014, household LLIN coverage was 83 %; 80 % of participants reported sleeping under a net the previous night. Mean malaria prevalence was estimated to range between 22 and 25 % after the short and long rains, respectively, and was significantly higher among children aged 5–14 years old, compared to those <5 years old (38 % vs. 18 % and 39 % vs. 34 % after the short and long rains, respectively).

**Fig. 1:**
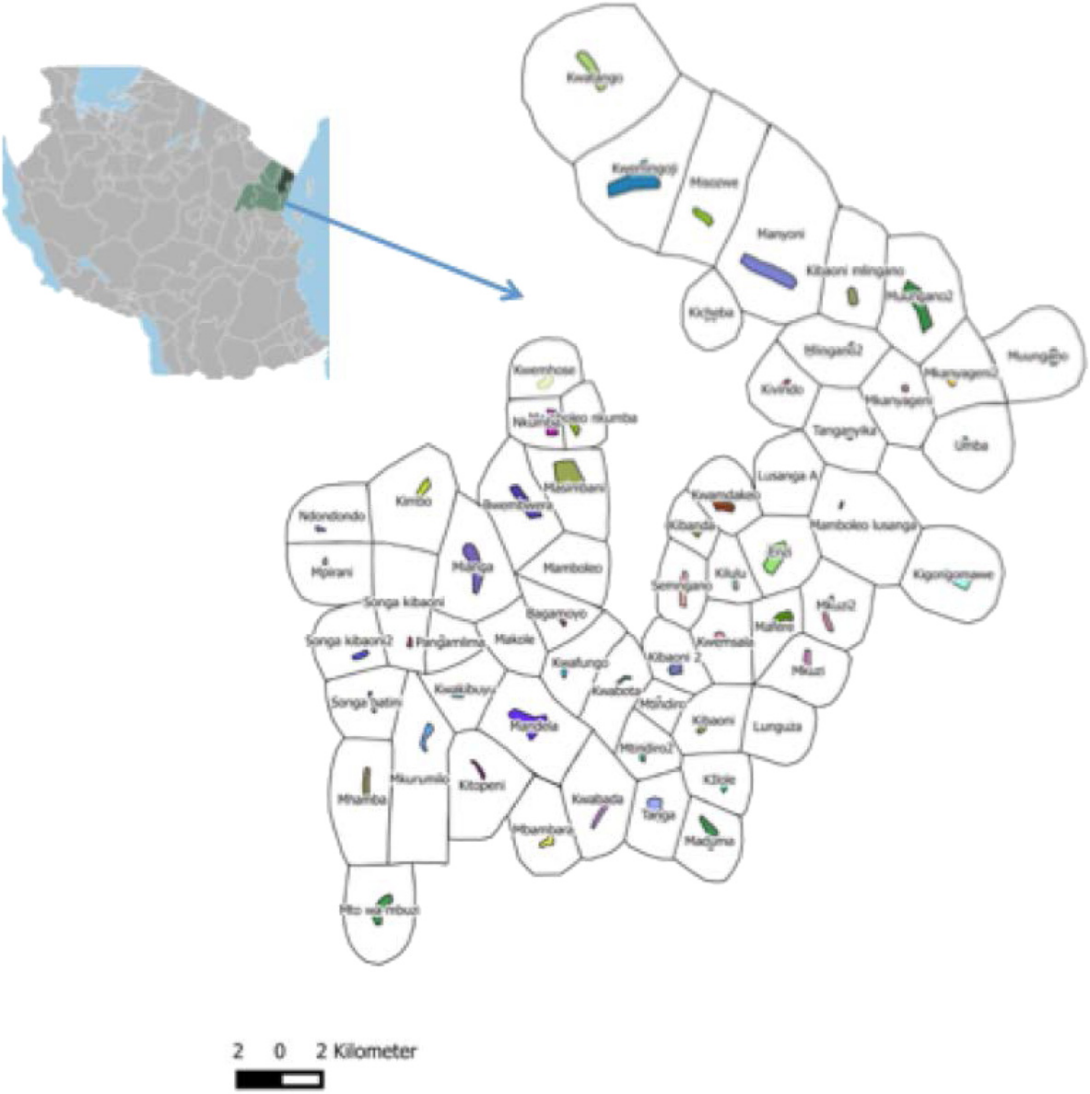
Map showing 60 candidate study clusters identified in Muheza District, including core (coloured) and buffer (*white*) areas

Villages or groups of neighbouring villages that have >125 children aged 6 months to 11 years old will be selected to form study clusters. Adjacent clusters will be ≥2 km apart to reduce mosquito contamination between study arms and maximize potential community effect of the interventions (Fig. 1). Other selection criteria include willingness to participate, road accessibility, proximity to the Muheza District hospital, the National Institute of Medical Research (NIMR) laboratory and insectary facilities of Amani Medical Research Centre and with no other ongoing malaria interventions at time of recruitment. Each cluster will consist of a central ‘core’ area where the study outcomes will be measured and a surrounding ‘buffer’ area of approximately 1 km (minimum of 2 km between adjacent core areas) where the same interventions will be implemented but no surveys undertaken. It is expected that each cluster will contain a minimum of 275 households and each core area will have a minimum of 124 households. It is assumed that each household will have approximately one child aged between 6 months to 11 years.

The study cohort of village children aged 6 months to 11 years old, who live in core cluster areas, will be enrolled to assess the effectiveness of the interventions on malaria parasitaemia (asymptomatic and symptomatic), the prevalence of anaemia and the costs associated with malaria infection and treatment. One hundred and ten children per cluster will be randomly selected and followed monthly for 1 year after intervention. No distinctions will be made regarding gender, ethnic group, medical condition or physical health. For households with multiple, eligible children, all potential participants will be recruited. Children will only be included in the cohort provided their parents/guardians give witnessed informed written consent or in the case of older children, personal assent is obtained. If consent is not provided then replacement children will be randomly selected from those remaining in the cluster. Assurance will be sought from parents/guardians that their child will remain resident in the village throughout the study year. Participants and households are free to withdraw from the study at any time with impunity.

### Design

A parallel cluster randomized controlled study design will be used as ITWL is a community-level intervention and a village cluster is a suitable unit for randomization. The study monitoring period will be 12 months, beginning December 2015, which will encompass one long (February-May, 2016) and one short rainy season (October–December, 2016) and two intervening dry seasons (Table 1). Following household enumeration, baseline socio-demographic, economic and household construction data will be collected. At study onset, all participating households will be provided with UC of LLINs, defined as one LLIN per every two persons. Clusters will then be randomized into two equal groups; all households in villages from one group will receive ITWL installed on interior house walls.

**Table 1:**
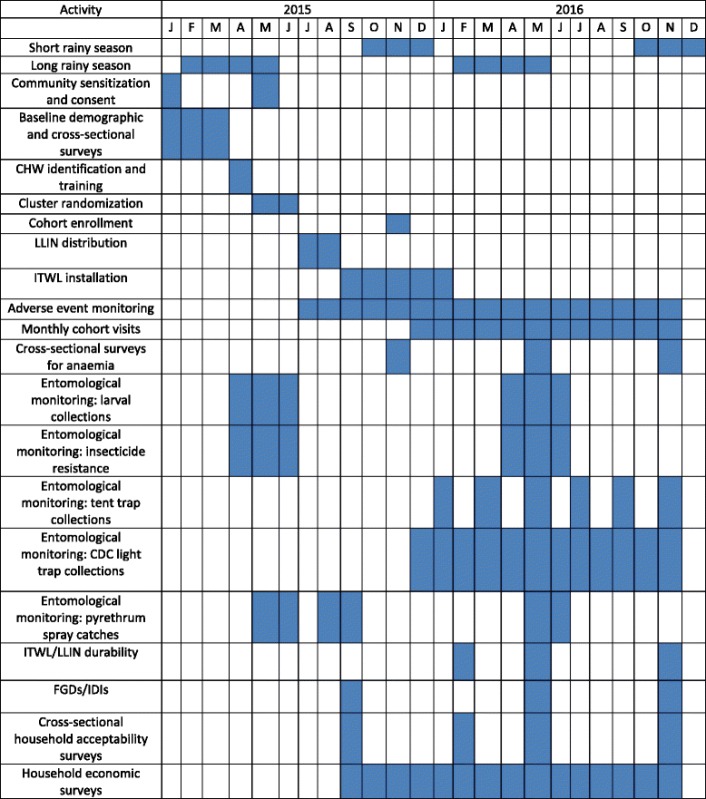
Timetable of study activities

To assess whether ITWL and UC of LLINs combined provide more protection against malaria in children than LLINs alone, a cohort of children aged 6 months to 11 years old from cluster cores will be followed monthly over 1 year. Malaria infection will be detected by malaria rapid diagnostic test (mRDT; One Step Malaria HRP-II (P.f) and pLDH (Pan) Antigen Rapid Test, Standard Diagnostics Inc., Korea) and positive children will be treated according to national guidelines. This cohort will also be surveyed for moderate (haemoglobin 7–8 g/dl) to severe anaemia (haemoglobin <7 g/dl) at enrollment, 6 months and 1 year post-intervention.

Exposure to malaria vectors will be measured indoors using Centers for Disease Control and Prevention (CDC) miniature light traps every month in eight randomly selected household in 12 study clusters/arm and outdoors using Furvela tent traps bi-monthly, in five randomly selected clusters/arm. Mosquito collections will be identified to species level and analyzed for sporozoite infection, *kdr* mutation status and blood meal source. ITWL bioefficacy will be monitored using WHO cone and cylinder bioassays and durability via High Performance Liquid Chromatography (HPLC) analysis and observational household surveys at three, six and 12 months post-intervention.

Household and community acceptability of ITWLs and LLINs will be evaluated in a series of focus group discussions (FGDs), in-depth interviews (IDIs), informal community observations and using a quantitative acceptability questionnaire before intervention and periodically throughout the monitoring year. To estimate the cost-effectiveness of ITWL and LLINs vs. LLINs alone, interviews will be conducted with residents who had a malaria incident 30 days prior, to determine the direct and indirect costs per malaria case. The cost of the intervention will be the net cost of installing ITWL and cost of malaria episodes in the study areas. The effectiveness will be the number of malaria cases averted across the age spectrum in the intervention areas, which will be assessed by collecting supplementary data from an ‘older’ sub-set of participants (≥12 years old), and will be compared to the control areas which only received LLINs.

**Fig. 2:**
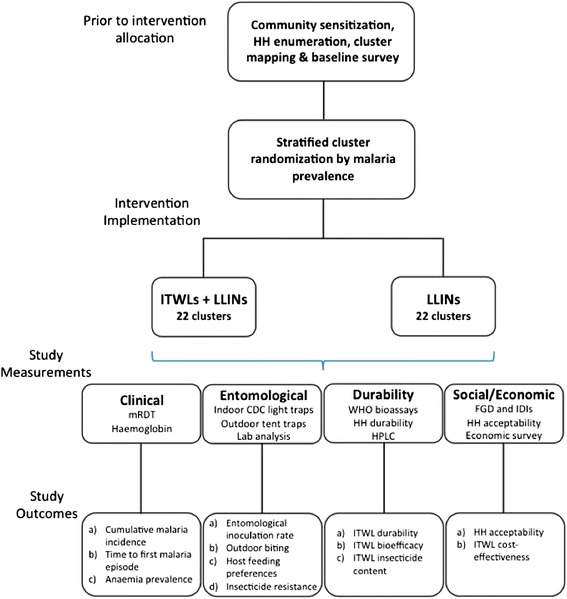
Schematic of study design

A schematic representation of the trial is shown in Fig. 2.

### Randomization and blinding

In cluster randomized controlled trials it is particularly important to minimize imbalance for factors known to be highly correlated with disease outcome, in this case house construction/presence of open eaves and LLIN usage. Since all households will be provided with LLINs and the majority of homes have open eaves (89 % during baseline surveys), clusters will be stratified into two groups based solely on malaria prevalence; clusters in each stratum will then be randomly assigned to the two study arms. Stratified randomization of villages will reduce the likelihood of chance imbalances between study arms but as only a relatively small number of units can be randomized in such a cluster design, both groups cannot be assumed comparable for all factors. Baseline data on malaria prevalence and haemoglobin concentration collected from all enrolled cohort participants will be used to assess disparity in disease burden at the village level prior to study monitoring.

Observer bias will be reduced where feasible; because of the visible and obvious nature of the intervention, it is not possible to conduct ITWL installation in a blinded manner. Mosquito collector bias will be reduced by using standard CDC miniature light traps and Furvela tent traps, which do not rely on the technical ability of the fieldworker to collect specimens. Trap catches will be examined by a different person to the trap collector and they will be blinded to the trap location and intervention status. The intervention implementation, for both ITWL installation and LLIN distribution, will be monitored closely for quality and to document any bias between the clusters. Epidemiological data entry will be conducted using smartphones to minimize errors in collection; GPS location and time of questionnaire administration will also be recorded to ensure that interviews are appropriately conducted and adequate time has been devoted to data collection.

Loss to follow-up will be minimized through the use of community health workers (CHWs) from each cluster to ensure children are available during monthly study visits. In the event of missing participants, the study team, assisted by CHWs, will undertake home visits to locate cohort members or may extend the monthly visits to each cluster to sample participants who are temporarily unavailable. Extended periods of time spent living away from a participant’s cluster will be recorded and factored into the analysis.

### Study interventions

**Fig. 3:**
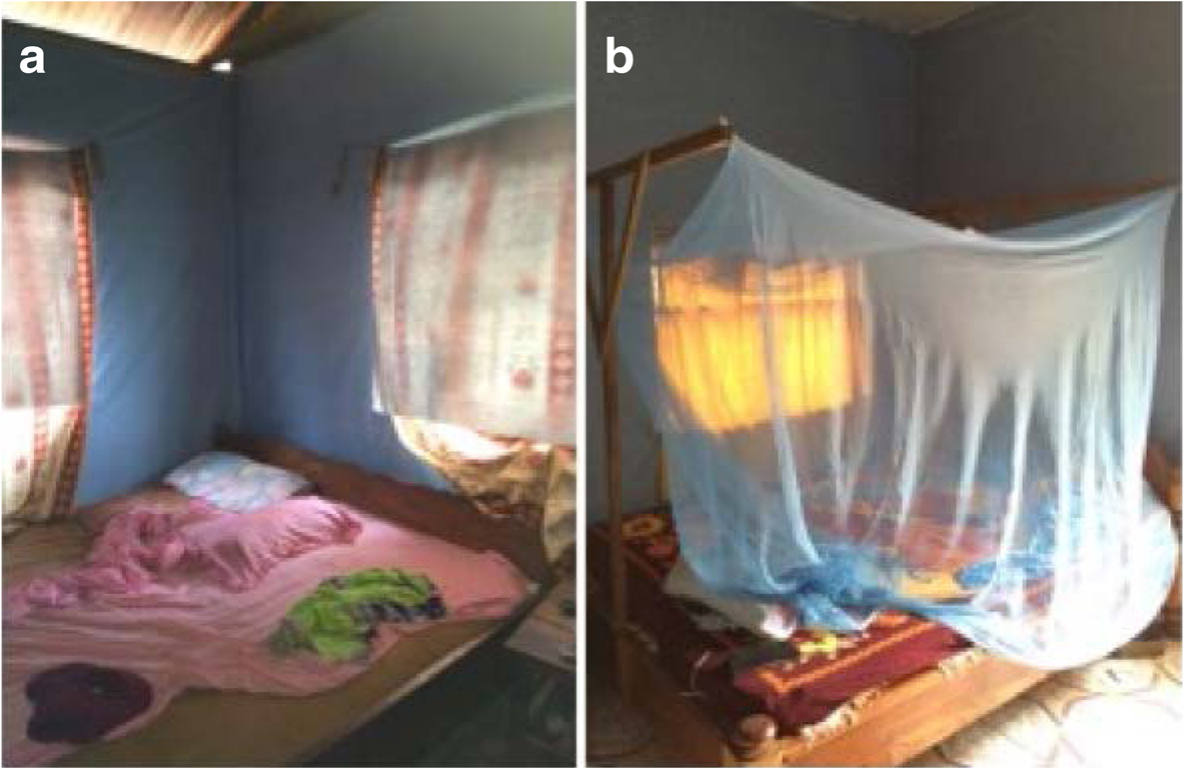
**a** PermaNet® Lining. **b** Interceptor® LLINs
- Long­lasting insecticidal nets (LLINs)
  All LLINs distributed during this study will be WHO-approved blue rectangular (160 cm wide x 180 cm long x 180 cm high) Interceptor® nets (BASF Corporation, Germany), which contain alphacypermethrin (200 mg/m^2^) coated onto polyester fibres [41] (Fig. 3b).

- Insecticide­treated wall lining (ITWL)
  In the ITWL plus LLINs study arm, ITWL installation will begin before the start of the short rainy season in late August 2015. PermaNet® Lining (Vestergaard Frandsen, Switzerland) is a high­density polypropylene fabric containing a proprietary combination of two non­pyrethroid insecticides (Fig. 3a), which is intended to mitigate potential development of insecticide resistance. One side of the fabric, designed to be attached directly onto house walls, is UV and moisture resistant, dust free and thermostable; the other side, which faces the house interior contains the insecticidal mixture incorporated into the polymer.

### Study intervention implementation

Prior to intervention implementation, a tiered and repetitive approach will be taken to sensitize the community to the objectives of the trial. Letters of introduction will be sent to the District Medical Officer (DMO) and District Executive Director of Muheza who will then introduce the research team to the selected study villages. Meetings will be held with these district officials, village-cluster and sub-village leaders to inform them of the study, explain the procedures and timelines and to address any questions or concerns. Because new issues can arise at any point, study staff will regularly attend community meetings throughout the monitoring period to address any problems and to provide an update of study progress.

Distribution of LLINs will be timed to occur shortly before ITWL installation in July-August, 2015, to ensure that all study households are protected before the beginning of the short rains and that participants are actively using their LLINs prior to ITWL intervention. LLINs will be distributed house-to-house, where one net will be given per every two persons. House members will be given instructions on proper use and maintenance of nets by study personnel.

ITWL installation is scheduled to begin in all buffer areas in late August 2015, to allow study teams the opportunity to remedy any unforeseen technical or logistical problems before installation in core households. ITWL installation will be rolled out in tandem with cohort recruitment. The order in which intervention clusters will receive ITWL installations will be randomized; once all eligible cohort members have been recruited in an intervention cluster and in a parallel randomly selected control cluster, ITWL will be installed in all households in the core area of the intervention cluster.

ITWL will be installed in each house by a three person team. Each roll of ITWL is blue, measures approximately 2.4 m × 210 m, and will be cut to fit specific house or room dimensions, recorded on the day of installation. During installation, after all pieces of furniture are moved into the centre of the room and wall items removed, the team will work around the room fixing the ITWL to all interior wall surfaces with nails at regular 60–70 cm intervals; the material will be cut around doors and windows and all eaves will be left uncovered. In houses with multiple rooms, ITWL will be installed in sleeping and living areas only; kitchens, toilets and storage room walls will remain bare. Educational material including information about maintenance and cleaning of ITWL will be distributed to householders. The study team will not make any formal repairs or install ITWL in new village houses once the monitoring period has begun.

### Clinical evaluations and patient treatment

Incidence of malaria parasitaemia (asymptomatic or symptomatic) will be measured among the study cohort monthly for 1 year by teams, each consisting of an experienced nurse or clinical officer and a CHW. Following informed consent, at house-to-house enrollment, socio-demographic and economic information, including age, sex, education, sleeping habits, mosquito net use, household wealth, migration and health care-seeking behavior will be collected using a standardized questionnaire. The clinical team will initially assess each child for any severity signs and referrals will be made for those with severe criteria. Each child will then undergo a clinical assessment, including a full medical history and physical examination (weight, mid-arm circumference for children <5 years old, height, respiratory rate, pallor and temperature), and blood will be collected by finger-prick to perform an immediate mRDT among all cohort members and to measure haemoglobin concentration using a HemoCue® spectrophotometer (AB Leo Diagnostics, Helsinborg, Sweden) in those aged 6 to 59 months.

At enrollment, all children will receive a curative weight-appropriate three-day course of artemether-lumefantrine (ALU), according to the Tanzanian National Treatment Guidelines. Baseline treatment is intended to clear any parasitaemia, irrespective of symptoms, considering recent data suggest >50 % of malaria patients confirmed by PCR are asymptomatic and negative by mRDT or microscopy [42], and ALU provides a prophylactic effect, thus reducing the likelihood that a cohort member will acquire infection prior to the intervention. Adherence to the first ALU dose will be observed by the study team at enrollment and parents/guardians will be instructed to administer the subsequent doses. Regimen completion will be checked by CHWs and verified at the first month visit by production of the empty drug packet. Families whose children are enrolled will be issued an enumerated identify card containing participant number and village and the scheduled dates for subsequent visits.

Active monthly follow-ups will occur at village sentinel sites. CHWs will be responsible for organizing village leaders and parents in advance of the scheduled visit, including arranging for school-aged children to be monitored outside school hours. At each follow-up, the clinical team will administer a physical examination, as described above, and record a short medical history to assess recent febrile illness, LLIN use and health care-seeking behaviour. A blood specimen will be collected by finger-prick for an immediate mRDT; anaemia (moderate: haemoglobin 7–8 g/dl to severe: haemoglobin <7 g/dl) will be measured in children aged 6 to 59 months at enrollment, at 6 months and 1 year post-intervention. In the case of a positive mRDT, the child will receive a curative weight-appropriate course of ALU, unless the child is already taking an appropriate antimalarial or has been within the past 2 weeks.

During the course of the study, the clinical team will also provide treatment for non-malaria illnesses, including paracetamol if axillary temperature is ≥37.5 °C, amoxicillin for non-severe pneumonia, oral rehydration salts (ORS) plus zinc for gastroenteritis and ferrous sulphate plus albendazole for anaemia (haemoglobin <9 g/dl). Children with severe illness at any time will be referred to Teule district hospital for further management. Participants are also free to receive medication from health personnel outside of the study teams.

Enrolled cohort children will be provided separate study booklets that their parents or guardians will be instructed to present each time that their child visits a health facility during the follow-up period. The 24 health facilities serving the study area will be visited by the study teams prior to the start of the study. Healthcare workers will be trained to properly complete the study booklet for enrolled children, recording whether the child was tested for malaria either by microscopy or mRDT during the visit, the result of the test, and what medication was prescribed or administered. During the monthly follow-up visits, study teams will ask parents or guardians of enrolled cohort children to present the study booklets, and any data on interim health facility visits will be collected electronically. Parents and guardians will also be asked to describe any other illness episodes that were not recorded in the study booklet.

### Entomological evaluations

The primary entomological endpoint (EIR) will be measured using CDC miniature light traps in eight randomly selected households with open eaves in 24 core cluster areas (12 clusters per study arm), every month (Table 1) [43]. Eligible households will contain children enrolled in the cohort study. In each household a CDC miniature light trap will be positioned 50 cm away from a single sleeper protected by a LLIN. To compare the extent of outdoor biting and transmission, every other month Furvela tent trapping will be undertaken in coordination with indoor light trapping [44]. One tent will be used in each cluster per night, set close to one of CDC light trapping households. Indoor and outdoor mosquito collections will be identified to species complex by microscopy [45] and the numbers of *An. gambiae* s. l., *An. funestus* s. l. and other *Anopheles* recorded. In each trap collection, a subset of *An. gambiae* s. l*.* and *An. funestus* s. l. will be speciated by PCR [46, 47]. In the case of *An. gambiae* s. l. knockdown resistance status (presence of *kdr* L1014S or L1014F alleles; previously *kdr-*east or *kdr*-west) will be determined by PCR [48]. *Plasmodium falciparum* sporozoite infection rates in *An. gambiae* s. s*., An. arabiensis* and *An funestus* s. l. will be assayed using a circumsporozoite protein (CSP) enzyme-linked immunosorbent assay (ELISA) [49].

To compare indoor mosquito resting density between the two study arms, early morning pyrethrum spray catches (PSCs) will be conducted in one household per cluster (total of 48 households). PSCs will be performed before intervention (during the long rainy season in May/June 2015), once during the cool dry season (August/September 2015) and during the second long rainy season (May/June 2016) (Table 1). To determine host feeding preferences, all blood fed malaria vectors will be squashed and dried onto filter papers and blood meal sources identified by ELISA [50].

Insecticide resistance profiles in both study arms will be compared before (during the long rainy season in May/June 2015) and after intervention (during the second long rainy season in May/June 2016) using three different bioassays: WHO cylinder bioassays for resistance to pyrethroids [51], CDC bottle bioassays for resistance to the two non-pyrethroid ITWL active ingredients [52] and resistance to pyrethroids. All assays will be performed using locally collected field populations of *An. gambiae* s. l. and *An. funestus* s. l. Larvae will be collected by sampling multiple breeding sites from at least six clusters per study arm, and reared to adults in the NIMR insectary; all bioassays will be performed using 2–5 day old unfed adult females.

Resistance to alphacypermethrin and permethrin will be measured using WHO cylinders at standard diagnostic dosages [53]. In lieu of WHO cylinder bioassays, CDC bottle bioassays will be used to determine the 50 and 99 % lethal doses (LD_50_ and LD_99_) to the two non-pyrethroid ITWL active ingredients. To measure intensity of pyrethroid resistance, the susceptible reference strain *An. gambiae* s. s. Kisumu will be exposed to a range of alphacypermethrin and permethrin dosages (0.001 μg/ml–0.5 μg/ml) to determine the LD_50_ [54]. A range of dosages will then be chosen to test against wild collected adult *An. gambiae* s. l. and *An. funestus* s. l (and F1 generations) that kill between 10 and 100 % before calculating the LD_50_ and comparing the resistance ratios to the reference strain. The intensity of resistance tests will be repeated on field collected specimens at the end of each transmission season. For all WHO resistance tests, six replicates of 20 mosquitoes and one control replicate will be assayed and percent knockdown will be observed every 15 min during 1 h of exposure; mortality will be recorded after 24 h. Surviving and dead mosquitoes will be analyzed for species identification and *kdr* resistance status, as described above.

### Intervention monitoring: ITWL

Prior to ITWL installation, quality control of the ITWL material will be assessed using WHO cone [55] and modified WHO cylinder bioassays [51] from a random sample of four rolls from each of five production lots (20 rolls total). From each ITWL roll, three 25 × 25 cm squares will be cut from a 2.5 × 1 m section for testing. Four WHO cone assays will be tested per ITWL sample for a total of 12 cone assays per roll. WHO cones will be fixed to the ITWL sample with frames and ten 2–5 day old unfed susceptible *An. gambiae* s. s. Kisumu mosquitoes or wild collected adult *An. gambiae* s. l will be exposed for 30 min; knockdown will be recorded after 1 h and mortality after 24, 48 and 72 h; the same experimental conditions will be used for WHO cylinder bioassays.

ITWL durability surveys will be conducted in 5 randomly selected households from eight intervention clusters (total of 40 households) at three, six and 12 months after intervention. At each follow-up, interviewers will survey the house to evaluate the condition of the ITWL installation and to record failed fixings, material tearing, causes of damage, proportion of walls still covered with ITWL and any evidence of ITWL repair, maintenance or attrition. In the same houses, ITWL in situ bioefficacy will be assessed using five WHO cone bioassays conducted as described above, at two wall heights (<1 m and >1 m from the floor) for a total of 10 bioassays per house. In addition, 5 × 5 cm samples of ITWL will be cut from house walls and insecticide content measured using HPLC according to the Collaborative International Pesticides Analytical Council (CIPAC) protocol.

### Intervention monitoring: LLINs

As part of the standardized questionnaire administered during each monthly cohort follow-up, LLIN coverage, use and attrition will be monitored. Participants will be asked about number of LLINs owned and number of sleeping places covered at the household level using a modified version of the WHO LLIN durability questionnaire [56].

### Social acceptability and economic evaluations

To identify social, cultural and other factors that may affect acceptability and uptake of ITWL and LLINs in the study area, a series of FGDs and IDIs will be undertaken. Four FGDs will be conducted in two randomly selected villages from each study arm prior to intervention and in the same villages after ITWL installation and LLIN distribution (total of 16 FGDs). FGDs will be conducted with small groups (8–12 participants) of men and women separately to reflect the different roles played in Tanzanian society and to ensure that all members will feel able to contribute to the discussion. In each village, one FGD will comprise heads of households and the other mothers of children <5 and mothers of children aged 5 to 11 years old. FGDs will be led by a trained moderator who will use a guide with open-ended questions to explore with the group a series of broad topics relating to malaria, disease prevention, ITWL concept and installation, LLIN use and healthcare seeking behavior and access. FGDs will be digitally recorded with consent from participants and a note-taker will document all non-verbal communications.

IDIs will be conducted with village or hamlet leaders, CHWs from village health facilities and ITWL installers to explore the opinions of key individuals who may play important roles in influencing community members’ perceptions of ITWL and LLIN use. Four IDIs will be conducted per study arm prior to intervention and with the same individuals after ITWL installation and LLIN distribution (total of 16 IDIs). The IDIs will cover a range of similar topics to the FGDs. IDIs will be conducted by a trained interviewer and digitally recorded with participant’s consent; non-verbal communication will be documented by hand. Informal community observations relating to major study activities, including community sensitization meetings, ITWL installation and LLIN distribution, will also be recorded by the study teams. Following the FGDs and IDIs, the main themes identified will be used to design a standardized, closed-ended quantitative acceptability questionnaire to be administered at three, six and 12 months post-intervention.

To assess the cost-effectiveness of ITWL + LLINs vs. LLINs alone, supplementary data will be collected from an additional ‘older’ sub-set of participants (≥12 years old) who occupy the same households as members of the main study cohort. From the baseline census, it is estimated that the 110 children/core area will represent 70 households and each core household will contain an average of three ‘older’ individuals. To determine the incremental effectiveness of ITWL across the entire age spectrum, during monthly cohort follow-ups, the key informant will report whether within the preceding 30 days, any ‘older’ household member(s) experienced a febrile illness and what, if any, treatment was received; if the informant themselves report a febrile illness, they will be asked to be tested with an mRDT immediately.

To estimate the economic cost per malaria episode, macro-cost analysis of hospital statistics from Teule Hospital and Mkuzi Health Centre (Muheza’s only district hospital and major health centre, respectively) will be used to determine the medical costs (cost per bed day and per outpatient visit) associated with an episode of clinical malaria and a household economic survey will measure additional out-of-pocket expenditures associated with care-seeking, non-medical direct and indirect costs and the impact of malaria on household food security. The economic questionnaire will be conducted on a monthly basis among all hospitalized cases, identified during the cohort visits, and an additional, randomly selected 14 % of households containing cohort children, to detect malaria cases treated in other settings (e.g. ambulatory, treated at home or with no treatment). During the economic questionnaire, all household members who report a febrile illness during the preceding 30 days will be interviewed and if they were not tested for malaria, they will be asked to take an mRDT; for children under 18 years old, the mother will be interviewed on their behalf. Any reduction in malaria-related school absenteeism by cluster will be measured during the monthly cohort visits, relative to the number of days two local schools was operational (verified by trusted community leaders and/or head teachers).

To measure the cost of LLINs distribution and ITWL installation, a micro-costing or ingredient approach to determine the personnel effort and materials, and a top-down costing approach to estimate the indirect cost associated management, oversight, transportation, and monitoring will be used. Additional meta-data on the cost of IRS implementation in other Tanzanian districts will be provided by Research Triangle Institute (RTI) and recently published estimates of the effectiveness of IRS in combination with LLINs [5], will be compared with the cost-effectiveness of ITWL.

### Trial oversight, safety considerations and handling withdrawals/drop-outs

Trial oversight will be provided by a Data and Safety Monitoring Board (DSMB) comprising three independent clinical and epidemiological experts. The role of the DSMB is to safeguard the interests of study participants as well as assess the safety and effectiveness of the study interventions. Independent data reviews by the DSMB will be conducted every 3 months throughout the study year.

Any adverse events (AEs) arising from either LLINs or ITWL will be closely monitored and documented. Interceptor® LLINs have been fully evaluated by the WHO Pesticide Evaluation Scheme (WHOPES) and approved for vector control; the product will be used in compliance with their recommended guidelines [57]. The non-pyrethroid insecticides in the ITWL have low mammalian toxicity and good safety profiles [58]. The proprietary combination formula used in the ITWLs has passed an initial environmental examination (IEE) conducted by an independent regulatory agency; hazard quotients for continuous habitation in a residence with ITWL were far below the acceptable threshold. All ITWL installers, who will receive the highest levels of exposure, will wear personal protective equipment (PPE; a minimum of gloves, long sleeves and eye protection) during intervention implementation. ITWL installers will undergo pre-installation, mid-way and post-installation medical examinations, which will include monitoring for any AEs.

During the ITWL intervention period, all core and buffer households will be visited 6–10 days following installation to document any AEs. Adverse event monitoring will also occur among ITWL installers through a passive AE monitoring chain. Throughout the study year, AEs will be monitored actively in each monthly cohort follow-up visit, during which the clinical team will ask the parents of cohort participants to report all AEs for any members of their household. Cohort members will also be given a study card to take to local health facilities, which will be used to record any AEs arising between monthly study visits. Other study participants will report AEs at any point to the CHWs, health facility or study team through a passive AE monitoring chain in both core and buffer areas. All AEs such as skin or eye irritation, respiratory problems, dizziness, nausea etc., will be graded in terms of severity (mild, moderate or severe), seriousness (based on patient outcome) and relatedness to the intervention by comparison with the control arm [58]. Appropriate management for any AE will be provided according to local guidelines and the outcome of AEs, including resolution, persistent disability, birth defect or death will be recorded.

In the case of a serious adverse event (SAE), the study clinician will record and manage the SAE in accordance with Good Clinical Practice (GCP) and report these to the PI who will inform the responsible member of the trial DSMB. Excessive grouping of SAEs by village or cluster will be reported to the DSMB and respective institutional ethics committees. A determination by the DSMB that there is potential harm to participants or the environment caused by the interventions will result in discontinuation of the study.

If an individual or household wants to terminate their participation, no further follow-up will be performed. If a cohort participant misses three consecutive monthly follow-up visits, they will be excluded from the study analysis. There will be no participant replacement during the surveillance period. If a household withdraws that was participating in the durability monitoring, it will be substituted with a neighbouring house of similar construction, where possible; there will be no other household or village replacements.

### Study endpoints

#### Epidemiological

Primary endpoint: the cumulative incidence of malaria parasitaemia (asymptomatic and symptomatic) defined as the number of mRDT-confirmed episodes of parasitaemia per child per year. A child will be considered to have experienced an episode of parasitaemia during a given month if they had a positive mRDT during the scheduled monthly follow-up visit, irrespective of symptoms. Two consecutive positive scheduled mRDT results will be treated as two separate episodes and any malaria cases diagnosed and treated at health facilities between monthly visits will also be counted as episodes.

Secondary endpoints: (i) time to first episode of malaria parasitaemia (asymptomatic and symptomatic) as determined by mRDT as per the primary case definition; (ii) changes in mean levels of haemoglobin among cohort children aged 6 to 59 months.

All study outcomes will be compared between the intervention (ITWL + LLINs) and the control arm (LLINs only).

#### Entomological

Transmission parameters in the two study arms will be estimated from measurements made throughout the transmission season.

Primary endpoint: the EIR for *An. gambiae* s. l. and *An. funestus* s. l. estimated as the mean number of sporozoite infective bites/person/year.

Secondary endpoints: (i) the relative abundance of outdoor host-seeking *An. gambiae* s. l. and *An. funestus*; (ii) host feeding preferences of *An. gambiae* s. l.; (iii) changes in frequency (% mortality using diagnostic dose) and strength (LD_50_ and resistance ratio) of insecticide resistance to alphacypermethrin, permethrin, non-pyrethroid insecticides in each study arm; (iv) ITWL bioefficacy and durability over 12 months.

### Sample size rationale

#### Epidemiological

LLINs can reduce malaria morbidity by approximately 40–70 % [3]; the impact of ITWL alone or in combination with LLINs on malaria is unknown. The effects may be additive or synergistic (e.g. by having a mass killing effect and/or reducing mosquito longevity) or antagonistic (e.g. if the LLINs are repellent thereby reducing mosquito house entry and contact with ITWL). Due to the intensity and projected cost of the ITWL intervention, a minimum reduction in malaria of 30 % is considered of public health importance.

For the cohort study, sample size is based on annual cumulative incidence of malaria infection in each study arm as the primary outcome. From baseline surveys conducted in Muheza, it was assumed that cumulative incidence of malaria would be 2.5–3.0 detected episodes/year, with a coefficient of variation (CV) between clusters of 0.302 and an intra-cluster correlation (ICC) of 0.047. Therefore with 22 clusters per arm, sampling an average of 110 children (assuming 18 % loss to follow-up) per cluster, the study will have >85 % power to detect a 30 % reduction in malaria incidence at the 5 % significance level [59].

To measure time to first malaria episode during the evaluation year, assuming an ICC of 0.047, for a cumulative incidence of 2.0 episodes/year, the monthly risk would be 0.167. Using a binomial distribution calculation, the probability of having zero positive determinations (i.e. of ‘surviving’ malaria-free) over 1 year is (1–0.167)^12^ = 0.112; with a monthly 30 % reduction in risk, the cumulative probability of having no monthly positive RDTs in 12 months in the intervention arm would be (1–0.117)^12^ = 0.226. Sampling from 44 clusters with 90 children per cluster, the study will have 99 % power to reject the null hypothesis of no difference in ‘survival’.

To assess the effectiveness of the interventions on anaemia in children aged 6 to 59 months, assuming mean haemoglobin of 10.75 g/dl, 22 clusters in each study arm and a CV of 0.048, the study will be able to detect a difference of 0.5 g/dl in mean haemoglobin with 5 % significance and 85 % power between the study arms, sampling an average of 45 children per cluster.

#### Entomological

Monitoring of indoor mosquito density will be conducted bi-monthly in eight different randomly selected households from six clusters per study arms (total of 576 households). All anopheline mosquitoes from each household will be randomly chosen for analysis (speciation, sporozoite ELISA, *kdr* resistance status and blood meal source).

### Data handling and record keeping

All demographic and clinical data will be collected on Android smartphones containing pre-programmed, pre-tested, standardized data entry forms, and sent directly to an electronic server. Each cohort member will have a unique identification number (ID) and non-cohort participants will be identified by a demographic enumeration number. All forms and datasets will record participants using these codes; no personal identifiers will be entered.

All data computers will be password protected with restricted access to only authorized study investigators and data management staff; field workers will have no access to the server. Daily data checks will be performed to identify incomplete, missing, inaccurate or inconsistent data, which will be rectified by the appropriate study investigator. Clinical data will be kept separately from that containing personal information. Data will be stored for at least 10 years.

The PI will maintain appropriate medical and research records in compliance with GCP and regulatory and institutional requirements. Authorized representatives of the sponsor, the ethics committee(s), the DSMB or other regulatory bodies may inspect all documents and records at any time. The PI will ensure access to facilities and records, as required.

### Data analysis

All statistical analyses will be performed using STATA version 13 (StataCorp LP, College Station, TX, USA). The primary endpoint will be a comparison of the cumulative incidence of symptomatic or asymptomatic episodes of malaria in children between the two intervention groups. Data from cohort members who are lost to follow-up or who missed monthly visit(s) will be treated as censored. Similarly, children who are taking anti-malarial drugs, based on a positive mRDT, will be excluded in the analysis for the first 2 weeks following treatment. History of travel away from the study village will be recorded during monthly visits and time at risk will be censored for such periods. In addition, malaria cases in children who resided outside their cluster area for more than half of the elapsed study period at the time of illness will be excluded.

An initial analysis will compare the cumulative malaria incidence rates between the two study arms to assess the number of episodes of malaria parasitaemia (symptomatic, asymptomatic or combined) per child per year. A follow-up analysis will examine the time to first episode of clinical malaria. The rate ratio (RR) will be calculated by dividing the incidence rate in the intervention relative to the control arm. The effectiveness of ITWL + LLINs vs. LLINs alone will be obtained from the final analysis as 1-RR. Cox regression modeling will be used to investigate potential confounding such as season and age. A priori confounders will be included in the model and that the presence of effect modification or interaction terms will be explored. Both intention to treat (all persons randomized) and per protocol analyses (all persons who received the intervention) will be conducted. All analyses will be adjusted for within-cluster correlation using generalized estimating equations or random effects models with gamma frailty. In addition, missing data will be analyzed and multiple imputation used, if required.

Similarly, mean haemoglobin in 6–59 month olds will be measured and compared between the two arms.

Other outcome variables, including entomological indices, cost-effectiveness and acceptability of interventions will be compared between study arms accounting for any correlated observations within clusters or households.

Digitally recorded FGDs and IDIs will be transcribed and translated verbatim and the data analysis procedure will follow a bottom-up approach by coding the raw data line-byline. The coding will be done by two research assistants to ensure intercoder reliability. Themes and theoretical constructs will emerge and will be grouped together as per the objectives. This method reflects a more analytical approach and will borrow the iterative approach of grounded theory analysis [60]. Data will be organized by importing into new QSR Nvivo 10 workbook and filed into the ‘internals’ section. Transcripts will be coded in an ongoing process as data is collected whereby a coding template will be developed from initial transcripts. As more data is received and coded, the template will be further refined to reflect any new emerging ideas or themes. These will appear in Nvivo as nodes which will be arranged in groups under a parent node labeled with the theme. Beyond this, themes may be organized into wider groups representing theoretical constructs. Memos will be made to explain the justification behind forming the construct. The social scientists from the project together with the wider research team will develop a narrative linking the original research objectives with the participants’ subjective practices and experiences. The aim of these theoretical descriptions will be to reiterate the participants’ narratives in terms of the theoretical constructs.

Quantitative data derived from household acceptability questionnaires will be compared using appropriate summary statistics, such as mean differences and proportions. All exposure variables showing evidence of relationships with acceptability of the study intervention in univariate analysis will be fitted in a multivariable logistic regression model. A stepwise backwards strategy will be used to eliminate variables with no effect (p > 0.05), these multilevel models will adjust for clustering within village or household and investigate for possible confounding or interaction.

Economic data collected will be used to assess the direct and indirect costs per malaria episode averted in each study arm.

## Discussion

The future of malaria control in Sub-Saharan Africa is threatened by problems of consistent use, sustainability and the rapid expansion of pyrethroid-resistant vector populations. While reductions in disease burden achieved by LLINs and IRS are irrefutable, the development of novel vector control strategies and insecticide delivery mechanisms will be crucial to maintain gains and facilitate the movement towards malaria elimination and eradication.

This is the largest cluster randomized controlled trial to evaluate whether non-pyrethroid ITWL can provide additional protection against clinical malaria compared to the current best practice of UC with LLINs. The potential longer lifespan and residual activity of ITWL, the absence of repeated intervention costs, and community-wide effect may render ITWL a superior control technique to LLINs alone. ITWL is expected to deter indoor human biting and decrease mosquito longevity and abundance; together these effects should reduce malaria transmission intensity. Furthermore, the use of two non-pyrethroid insecticides over time may lead to partial reversal of the frequency and intensity of pyrethroid resistance among local vector populations [61].

The operational success of ITWL will be determined not only by its demonstrable protective efficacy but also its feasibility of implementation, levels of household acceptability, durability under field conditions and cost-effectiveness compared to other first-line strategies. This trial will investigate the effectiveness of ITWL in a ‘real-to-life’ community setting and aims to establish a precedent in multidisciplinary evaluations of new large-scale control interventions.

If ITWL is proven both effective, durable and feasible, it may warrant consideration for programmatic implementation. How ITWL would be executed to scale and in which particular epidemiological situations will depend on its relative cost-effectiveness compared to other main-line strategies (IRS and LLINs). ITWL is unlikely to supersede IRS as part of routine malaria control, but may have an important role to play as a mechanism of house improvement [62–64]. Alternatively, ITWL could serve as a complementary strategy for use in endemic transmission settings, particularly in areas where pyrethroid-resistant vectors predominate, or in regions moving towards malaria elimination [65, 66]. Trial findings will provide valuable information for the National Malaria Control Programme (NMCP) and policy makers in Tanzania and other malaria-endemic countries to guide finite resource allocations for future control efforts.

## Abbreviations

ACT, artemisinin combination therapy; AE, adverse event; ALU, artemether-lumefantrinev; CDC, Centers for Disease Control and Prevention; CHW, community health worker; CIPAC, Collaborative International Pesticides Analytical Council; CSP, circumsporozoite protein; CV, coefficient of variation; DMO, district medical officer; DSMB, data and safety monitoring board; EIR, entomological inoculation rate; ELISA, enzyme-linked immunosorbent assay; FGD, focus group discussion; GCP, good clinical practice; HPLC, high performance liquid chromatography; ICC, intra-cluster correlation; IDI, in-depth interview; IEE, initial environmental examination; IRS, indoor residual spraying; ITWL; insecticide-treated wall lining; KCMC, Kilimanjaro Christian Medical College; *kdr*, knockdown resistance; LD_50_, 50 % lethal dose; LLIN, long-lasting insecticidal net; LSHTM, London School of Hygiene and Tropical Medicine; MRCC, medical research coordinating committee; NIMR, National Institute of Medical Research; NMCP, national malaria control programme; PI, principal investigator; PPE, personal protective equipment; PSC, pyrethrum spray catch; RDT, rapid diagnostic test; RR, rate ratio; RTI, Research Triangle Institute; SAE, serious adverse event; UC, universal coverage; WHO, World Health Organization; WHOPES, WHO Pesticide Evaluation Scheme
