# Erratum to: The effectiveness of non-pyrethroid insecticide-treated durable wall lining to control malaria in rural Tanzania: study protocol for a two-armed cluster randomized trial

**DOI:** 10.1186/s12889-016-3856-5

**Published:** 2016-11-25

**Authors:** George Mtove, Joseph P. Mugasa, Louisa A. Messenger, Robert C. Malima, Peter Mangesho, Franklin Magogo, Mateusz Plucinski, Ramadhan Hashimu, Johnson Matowo, Donald Shepard, Bernard Batengana, Jackie Cook, Basiliana Emidi, Yara Halasa, Robert Kaaya, Aggrey Kihombo, Kimberly A. Lindblade, Geofrey Makenga, Robert Mpangala, Abraham Mwambuli, Ruth Mzava, Abubakary Mziray, George Olang, Richard M. Oxborough, Mohammed Seif, Edward Sambu, Aaron Samuels, Wema Sudi, John Thomas, Sophie Weston, Martin Alilio, Nancy Binkin, John Gimnig, Immo Kleinschmidt, Peter McElroy, Lawrence H. Moulton, Laura Norris, Trenton Ruebush, Meera Venkatesan, Mark Rowland, Franklin W. Mosha, William N. Kisinza

**Affiliations:** 1 National Institute for Medical Research, Amani Research Centre, Muheza, Tanzania; 2 Department of Disease Control, London School of Hygiene and Tropical Medicine, London, UK; 3 US President’s Malaria Initiative, Atlanta, GA USA; 4 Malaria Branch, Division of Parasitic Diseases and Malaria, Center for Global Health, Centers for Disease Control and Prevention, Atlanta, Georgia USA; 5 Kilimanjaro Christian Medical College, Moshi, Tanzania; 6 National Institute for Medical Research, Headquarters, Dar es Salaam, Tanzania; 7 Brandeis University, Heller School, Waltham, Massachusetts USA; 8 PMI Africa Indoor Residual Spraying Project, Abt Associates, London, UK; 9 Phoenix Ordinary LLC, Bridgewater, New Jersey USA; 10 President’s Malaria Initiative, United States Agency for International Development, Washington, DC USA; 11 Translating Research into Action Project (TRAction) University Research Co., LLC, Bethesda, Maryland USA; 12 Department of International Health, Johns Hopkins Bloomberg School of Public Health, Baltimore, Maryland USA

## Erratum

The authors of this published article [1] would like to correct the ‘Funding’ declarations to read as follows:

“This study was funded by the US President’s Malaria Initiative (PMI) through the United States Agency for International Development under Translating Research into Action, Cooperative Agreement No. GHS-A-00-09-00015-00, managed by University Research Co., LLC (URC).”
